# Differences between mitotically old and young endometrial tumors

**DOI:** 10.1002/path.70090

**Published:** 2026-07-08

**Authors:** Kellie Kim, Darryl Shibata

**Affiliations:** ^1^ Department of Pathology University of Southern California Keck School of Medicine Los Angeles CA USA

**Keywords:** mitotic age, endometrial adenocarcinoma, DNA methylation, epigenetic clock, tumor evolution, immune surveillance, T‐cell exhaustion

## Abstract

Human tumors likely differ in their mitotic ages, reflecting how many divisions elapse between the final tumor progenitor cell and surgical removal. We used a rapidly fluctuating CpG (fCpG) methylation clock to infer relative endometrial adenocarcinomas (EAC) mitotic ages. Experimentally, young tumors initiated from single cells show low‐diversity, high‐variance fCpG distributions with trimodal peaks near 0%, 50%, and 100%, reflecting inherited progenitor methylation states. fCpG methylation becomes polymorphic with divisions, and older tumors exhibit higher diversity, lower variance, and unimodal distributions centered around 50%. Mitotic ages varied across EAC samples. Synchronous hyperplasia and invasive regions generally shared similar ages, and primary–metastatic EAC pairs showed both synchronous and stepwise progression. The Cancer Genome Atlas (TCGA) EACs also showed variable mitotic ages: younger tumors were enriched for proliferation pathways, whereas older tumors showed more immune infiltration, immune‐pathway activation, and evidence of T‐cell exhaustion. These results show that human tumors can be ranked by mitotic age and suggest that the growth of older cancers is restrained by immune surveillance. © 2026 The Author(s). *The Journal of Pathology* published by John Wiley & Sons Ltd on behalf of The Pathological Society of Great Britain and Ireland.

## Introduction

**Figure 1:**
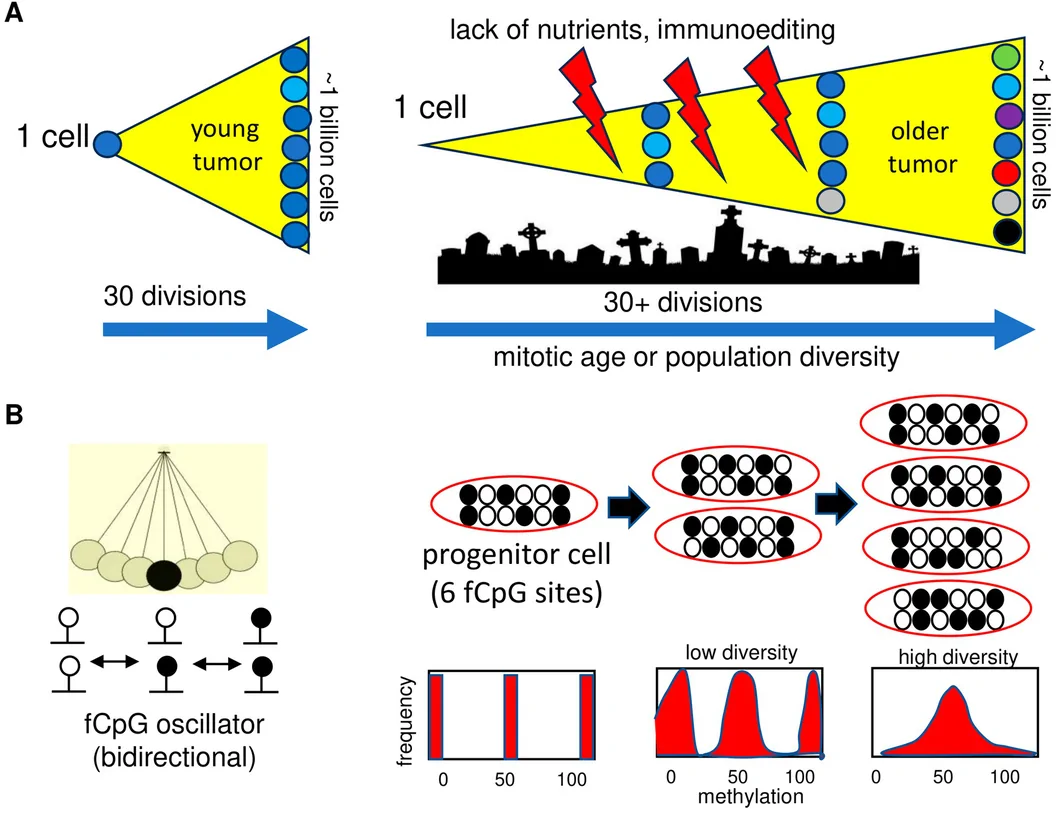
Higher diversity in mitotically older cancers. (A) The youngest cancer requires a minimum mitotic age of ~30 division for a single progenitor cell to grow to a detectable size of ~1 cm^3^ (~1 billion cells). Additional divisions are needed if tumor cells are lost during growth, either because the tumor microenvironment cannot sustain proliferation or because cells are eliminated by immunoediting. (B) Fluctuating CpG (fCpG) sites have oscillating but balanced methylation and demethylation, with average methylation ~50% in older, desynchronized, polyclonal cell populations. A fCpG clock is composed of hundreds of different fCpG sites and is reset at the start of growth by the pattern in the progenitor cell. fCpG methylation shows trimodal frequency distributions in new clonal expansions, with 0%, 50%, and 100% methylation peaks synchronized by the progenitor cell pattern. With more divisions, random replication errors progressively desynchronize methylation patterns between cells, leading to unimodal distributions around 50% methylation in older tumors. The accumulation of subclonal epimutations is captured by average methylation at each fCpG site, with maximal diversity near 50% methylation, making fCpG diversity a proxy for mitotic age.

Tumor mitotic age can be defined as the numbers of cell divisions between a final progenitor cell and tumor removal (Figure 1A). Tumor mitotic ages may differ, and there may be important clinical and biological differences between very young and older cancers. Understanding why some cancers require many more divisions before detection and removal may help identify mechanisms that naturally restrain tumor growth. The minimum age for a detectable tumor (a billion cells) is about 30 divisions (2^30^). Additional divisions are required if tumor cells are removed during growth, which may occur when the tumor microenvironment (TME) cannot sustain proliferation or when cells are actively eliminated by processes such as immunoediting [1]. Currently there are no robust methods to infer human cancer mitotic ages. Here we use a molecular clock approach to estimate population ages based on genomic diversity, under the principle that cells in older populations are more polymorphic (Figure 1B).

Human tumor evolution is commonly reconstructed from somatic mutations [2, 3, 4], but it is technically difficult to measure tumor genetic diversity because most detectable subclonal mutations (≥ 5% allele frequency) arise early, when the tumor is still very small [5]. For example, a new subclonal mutation in a tumor of only around 10^6^ cells would be present in < 0.0001 of the population and therefore undetectable by conventional sequencing.

Here we use the diversity of DNA methylation at fluctuating CpG sites (fCpGs [6]) to rank tumor ages. Although DNA methylation is widely used to infer human chronological age [7, 8], our approach differs because the fCpG clock resets to the progenitor's methylation pattern at the start of tumor growth, not at birth. This clock reset refers to the fCpG pattern synchronization in a new clonal expansion as the fCpG methylation in the progenitor cell is not reset to a starting state, but its current random state is the template of its progeny. Unlike unidirectional age‐associated drift, fCpGs undergo balanced bidirectional methylation and demethylation.

fCpGs are identifiable because their methylation is randomized between cells (population average ~50%) in older, polymorphic populations but shows trimodal, low‐diversity distributions in young clonal populations, with peaks near 0%, 50%, and 100% reflecting synchronization by the pattern in the founding cell (Figure 1B). Frequency peaks near 0% or 100% indicate low diversity because most cells share the same methylation state. With additional divisions, stochastic drift desynchronizes these patterns, and the population becomes progressively older and more polymorphic. A key advantage over mutation‐based approaches is that average fCpG methylation directly reflects underlying cell‐to‐cell diversity (maximal diversity at ~50%) and can be readily measured using standard methylation arrays; this study relies on suitable samples available in public databases.

First, we present experimental data [9] demonstrating that single‐cell subcloning resets the fCpG clock. Next, we use simulations to illustrate how fCpG methylation distributions can be used to infer relative tumor mitotic ages, and how fCpGs can function as dynamic barcodes to reconstruct ancestry between tumor regions. We then apply the fCpG clock to endometrioid adenocarcinoma (EAC), beginning with paired, synchronous hyperplasia and cancer regions previously inferred by sequencing to be related [10]. The clock is further used to infer the timing of metastasis in paired primary and metastatic EAC specimens [11]. Finally, The Cancer Genome Atlas (TCGA) EACs are ranked by mitotic age, and metadata [12] are used to compare biological differences between younger and older tumors.

## Materials and methods

### Identifying fCpG sites

fCpG sites have ~50% methylation in polyclonal populations and variable methylation in clonal populations of the same cell type, measured with Illumina 450k or 850k arrays [6]. The approach uses polyclonal and clonal samples. Average methylation at each CpG site is calculated among the samples, and candidate fCpGs have average methylation between 40% and 60% (sex chromosomes were excluded for the fibroblast clock and the Y chromosome was excluded for the EAC clock). fCpG site methylation should be variable between clonal samples, and fCpG sites are identified by ranking their variances between samples (which filters out CpGs with stable hemimethylation). For the EAC fCpG clock, variances at each fCpG between TCGA tumors varied between 0.13 and 0.03. Manual inspection is used to filter out CpG sites that behave like polymorphisms (or 0%, 50%, or 100% values in most samples, whereas fCpGs have a range of values between 0% and 100%). Typically, ~400 to 1,000 fCpG sites are used for each cell‐type‐specific mitotic clock (average methylation 40–60% between samples, and high variance between samples for each fCpG site). fCpGs show cell type specificity and suitable fCpG sites differ for each tissue. It is anticipated that fCpG clocks could be further improved with more formal optimization, including more samples, adjusting for biased methylation at some fCpG sites, and more detailed simulations. fCpG sites used for the fibroblast and EAC clocks are provided in supplementary material, File S1.

### fCpG clock analysis

Three values are calculated for each sample – average fCpG methylation across the clock sites, the variance of these methylation values, and epithelial cell purity estimated with LUMP [13]. Cancer samples with purity < 60% were excluded. For TCGA (https://www.cancer.gov/ccg/research/genome-sequencing/tcga) data, 24 samples had purity < 60% and 443 samples had purity > 60%. Although ideally each sample would have average fCpG clock methylation between 40% and 60%, some samples showed skewed averages outside this range but were retained in the analysis although they may violate underlying model assumptions. The proportion of samples with average fCpG clock methylation outside of the 40–60% range was 30% for TCGA samples, 21% for EAC‐hyperplasia samples [10], and 21% for primary‐metastatic samples [11]. Fewer samples were outside a broader 35–65% range, at 8%, 5%, and 3% respectively.

### Correlations TCGA metadata

TCGA data (array methylation and bulk RNA‐seq) were downloaded in 2024 from https://portal.gdc.cancer.gov/. A list of analyzed TCGA EAC samples used in this study is provided in the supplementary material, File S1. Metadata uniformly processed and analyzed by standard algorithms were from Thorsson *et al* [12] and were used without reprocessing. Pearson correlations were used to compare fCpG clock variances with available metadata and RNA‐seq features.

### fCpG clock simulations

Simulations were written in Python (https://www.python.org/) with the assistance of ChatGPT (https://chatgpt.com/) and are available by email upon request to corresponding author. The simulation starts with a single cell with about 500 fCpG sites, each initialized at 0%, 50% or 100% methylation. With each division, the mother cell can produce zero, one, or two daughter cells, and the pattern of the mother cell is copied to the daughter cell with a probability of alteration specified in the program. Growth rates are slowed by limiting the numbers of cells that produce two daughter cells; otherwise, cells produce only one daughter cell. Average methylation and population variance of the cells are measured at the end of growth. For the simulations in Figure 3, the final population sizes were about 100,000 cells with an average total methylation and demethylation rate of 0.0042 per CpG site per division. To better match the experimental data, heterogeneity in CpG flip rates was included, with error rates between 0.0004 and 0.01 per division, and slightly skewed bidirectional methylation and demethylation probabilities ranging from 0.39 to 0.61 (overall mean 0.5).

Stepwise progression was simulated by introducing a bottleneck (setting the probability of a daughter cell to 0) and removing most cells at the time of divergence. This population (one to eight cells) then grows to 100,000 cells at the time of removal, and its mitotic age (variance) and methylation pattern (Pearson correlation) are compared to the primary tumor.

## Results

### Single cell subcloning resets the fCpG clock

A fCpG clock consists of hundreds of CpG sites with oscillating, bidirectional, balanced methylation and demethylation [6]. Human intestinal data illustrate unimodal fCpG distributions in polyclonal bulk intestines, but trimodal fCpG distributions from individual clonal crypts. In addition, polyclonal blood shows unimodal distributions, whereas acute leukemias exhibit trimodal distributions and chronic leukemias have broader, more polymorphic distributions [6].

**Figure 2:**
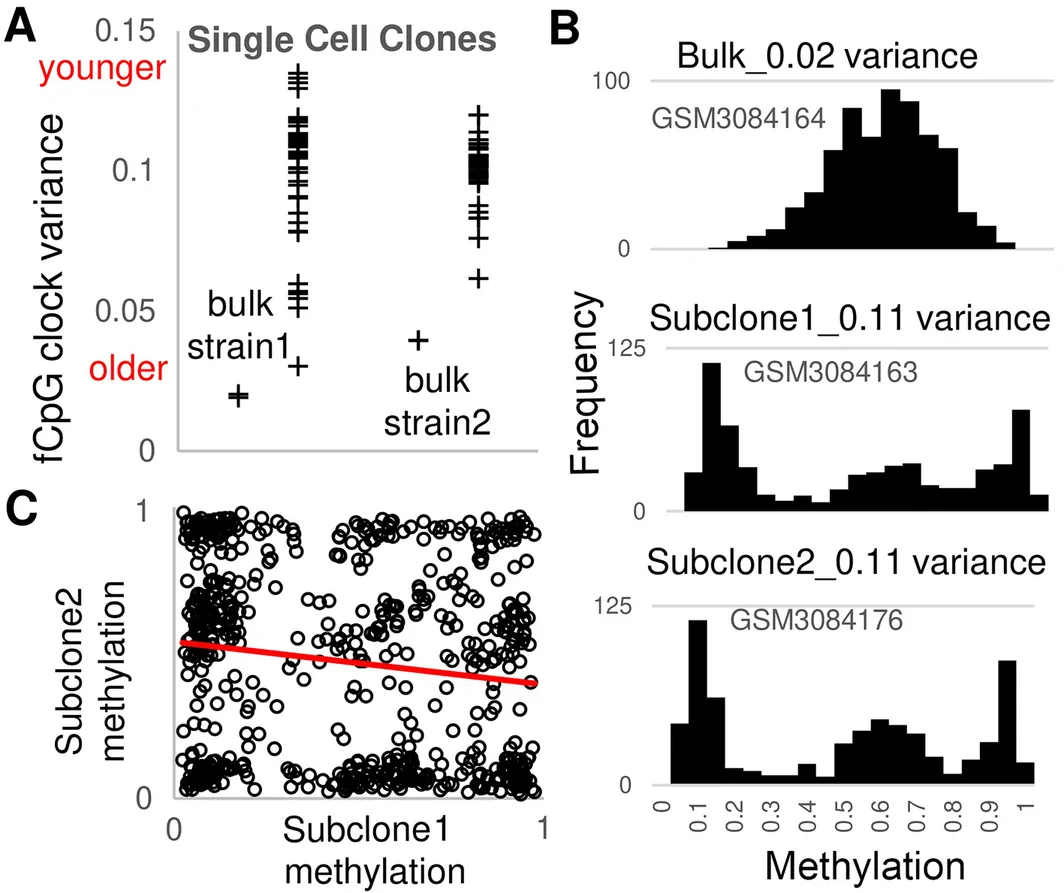
Single cell subcloning resets the fluctuating CpG (fCpG) clock. (A) fCpG clock variance is higher, indicating lower diversity and younger mitotic age, after single cell subcloning from two different human fibroblast strains (GSE11287 [9]). (B) fCpG clock methylation frequency distributions show high variance, trimodal ‘W‐shaped’ distributions in new subclones and unimodal low variance distributions in the parental polyclonal culture. Subcloning resets the fCpG clock to the pattern of the single cell progenitor. fCpG histograms of all fibroblast subclones and bulk samples are provided in supplementary material, Figure S3. (C) fCpG methylation comparisons between independent subclones reveal checkerboard patterns indicating that individual cells in the older, polyclonal bulk populations have diverse and desynchronized fCpG methylation.

The reset of a fCpG clock at the start of growth can be directly demonstrated with single cell subcloning. A new subclone should have a high variance fCpG distribution synchronized by the 0%, 50%, and 100% methylation pattern of its progenitor cell (Figure 2A). DNA methylation was measured from polyclonal and single cell fibroblast subclones (GSE112877 [9]). fCpG sites are cell type specific and are typically present in nongenic or in poorly expressed genes [6]. Consistent with expectations, the fibroblast fCpG clock with 2,000 fCpG sites shows unimodal distributions in polyclonal parental cultures and broader, often trimodal distributions in the single‐cell subclones (Figure 2B). fCpG methylation correlated poorly between many subclones, consistent with polymorphic fCpG methylation patterns among individual cells within the parental culture (Figure 2C). This experiment illustrates how a new clonal expansion started from a single cell resets the fCpG clock to the progenitor cell pattern (Figure 1B).

### fCpG clock simulations

**Figure 3:**
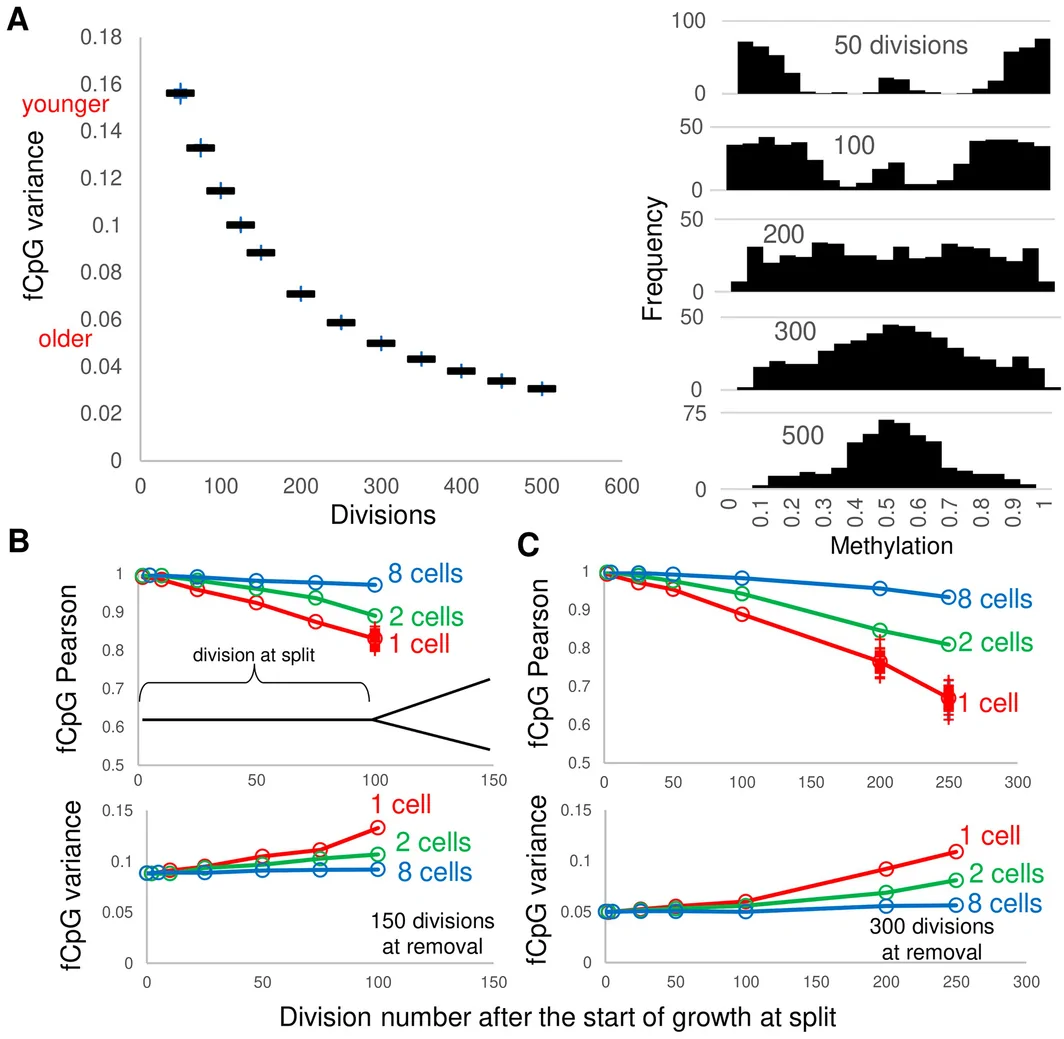
Fluctuating CpG (fCpG) clock simulations. (A) Simulations indicate that fCpG clock methylation variance decreases non‐linearly with mitotic age from high variance trimodal distributions in young populations to lower variance unimodal distributions in older populations. Data from 100 simulations at each time point are shown; bars represent mean values, with ‘+’ indicating individual runs, demonstrating low variability between simulations. (B) Simulated stepwise progression with different numbers of cells and divergence times. Two regions synchronously started from the same progenitor have similar mitotic ages (fCpG variances) and fCpG methylation patterns (high Pearson correlation). Differences in mitotic ages increase and fCpG methylation patterns become less correlated when divergence occurs later after the start of growth and when the new region is founded by fewer cells. The stepwise divergent tumor region is mitotically younger and less related to its primary tumor. Illustrated are simulations of a primary tumor reaching 150 divisions (with divergence at 25, 50, 75, or 100 divisions). There were 25 simulations for each data point, and variation between individual runs (‘+’) was only evident when simulated metastasis was founded by a single cell late in tumorigenesis. (C) Simulated stepwise progression for a primary tumor reaching 300 divisions (with divergence at 25, 50, 100, 200, or 250 divisions). Overall, fCpG patterns are more different (Pearson correlation and mitotic ages) between a primary and its simulated metastases when the seeding occurs stepwise with a smaller number of cells that migrate later after the start of its primary tumor.

The fCpG clock reset is simulated by starting with a single cell with hundreds of fCpG sites, each initiated at 0%, 50%, or 100% methylation. With every division, a cell can produce one, two, or zero daughter cells, and the methylation at each fCpG can randomly flip, with similar rates of methylation and demethylation. Older tumors are simulated by increasing the proportion of divisions that yield only one daughter cell, resulting in more total divisions before reaching detectable sizes. fCpG distributions start trimodal (high variance) and progressively become more unimodal (low variance) as cells become more polymorphic and diverse (Figure 3A). Thus, fCpG distribution variance serves as a proxy for mitotic age.

The fCpG clock can also infer ancestries of multiple samples from the same tumor. Samples are synchronous if they grow from the same progenitor, or stepwise when cells separate from an older primary tumor and grow into another detectable tumor. Simulations indicate that fCpG patterns are more different when stepwise progression occurs later in tumor growth and when it is founded by a small number (less than eight) of cells (Figure 3B,C). Stepwise progression is manifested by both mitotic‐age differences and lower fCpG Pearson correlation between regions.

These simulations indicate that mitotically younger cancers should exhibit high variance and trimodal fCpG distributions, whereas older cancers should have progressively lower variance and more unimodal fCpG distributions. Importantly, the fCpG clock infers mitotic ages; thus a tumor with a lower division rate may have a greater calendar age but the same mitotic age as a more rapidly dividing tumor. Greater fCpG pattern differences (both Pearson distances and mitotic ages) should be present between a primary tumor and its metastases when seeding occurs as a later stepwise event involving fewer migrating cells. While these simulations are limited to a narrow range of defined parameters and assume stable error and division rates that may not hold in all scenarios, they illustrate the fundamental fCpG clock mechanics: a single pattern in a progenitor cell synchronizes its new clonal expansion, which then becomes progressively polymorphic with further divisions.

### Synchronous EAC hyperplasia and carcinoma

EAC and hyperplasia are often in the same uterus, and one study of endometrioid tumors indicated that somatic mutations were similar between such lesions, consistent with synchronous rather than stepwise progression from hyperplasia to EAC [10]. This study also measured average CpG methylation with arrays (GSE136791; https://www.ncbi.nlm.nih.gov/geo/query/acc.cgi?acc=GSE136791), and a suitable fCpG EAC mitotic clock with 507 fCpG sites was developed with TCGA EAC data (see Materials and methods).

**Figure 4:**
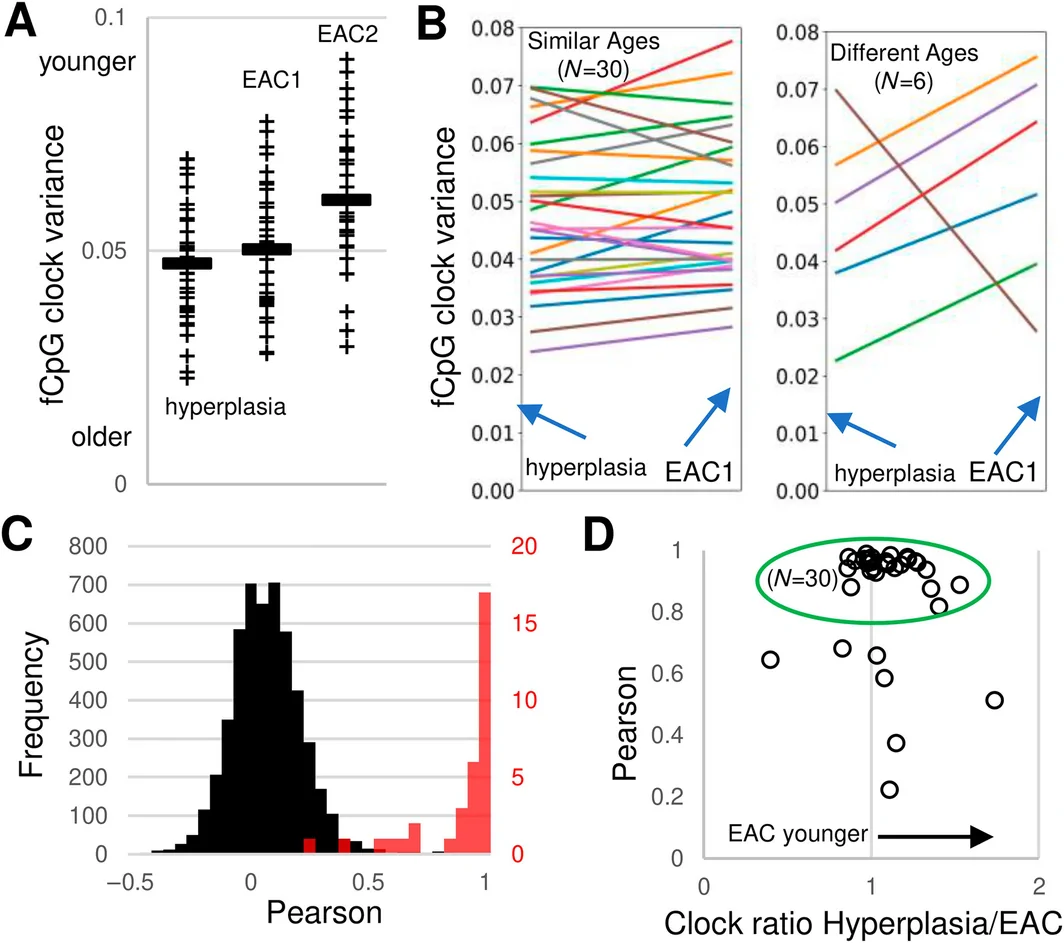
Synchronous endometrial adenocarcinoma (EAC) hyperplasia and cancer (GSE136791 [10]). (A) Fluctuating CpG (fCpG) variances (mitotic ages) differ between endometroid tumors with (EAC1) and without adjacent hyperplasia (EAC2). EAC2 without adjacent hyperplasia were younger than EAC1 with adjacent hyperplasia (*p =* 0.0074). Hyperplasia regions were older but the differences with EAC1 tumors were not significant (*p =* 0.56). fCpG histograms of each sample are provided in supplementary material, Figure S4. (B) Consistent with synchronous growth simulations (Figure 3B), fCpG variances (mitotic ages) were similar for most adjacent hyperplasia and EAC1 pairs. Pairs were considered similar if their fCpG variance ratios were near one (range 0.8–1.2). (C) fCpG patterns correlated poorly between unrelated EACs (average Pearson ~0), but consistent with synchronous growth, related hyperplasia and EAC pairs were generally more correlated (Pearson > 0.8). (D) Combining relative mitotic‐ages ratios (hyperplasia/EAC1 fCpG clock variances) and fCpG pattern similarity (Pearson correlation) reveals likely synchronous growth for most adjacent hyperplasia and EAC1 pairs (circled).

Inferred mitotic ages were different between tumors, with average ages of hyperplasia older than EAC1 tumors (with adjacent hyperplasia), and EAC2 tumors (without adjacent hyperplasia) younger than EAC1 tumors (Figure 4A). Consistent with synchronous growth, most (30 of 36) fCpG clock variances or inferred ages were similar between paired hyperplasia and EAC1 samples, with only one pair in which the hyperplasia appeared much younger than its cancer (Figure 4B). Also consistent with synchronous growth, fCpG methylation correlated well (Pearson correlation > 0.8) for most pairs (Figure 4C). Combining fCpG variances with fCpG correlations, 29 of 36 pairs most likely originated from the same single progenitor cell, showing similar ages and correlated fCpG patterns (Figure 4D). This dataset illustrates that EAC mitotic ages vary and is consistent with the previously inferred synchronous growth [10] for these adjacent hyperplasia–cancer pairs.

### Synchronous and stepwise metastases

**Figure 5:**
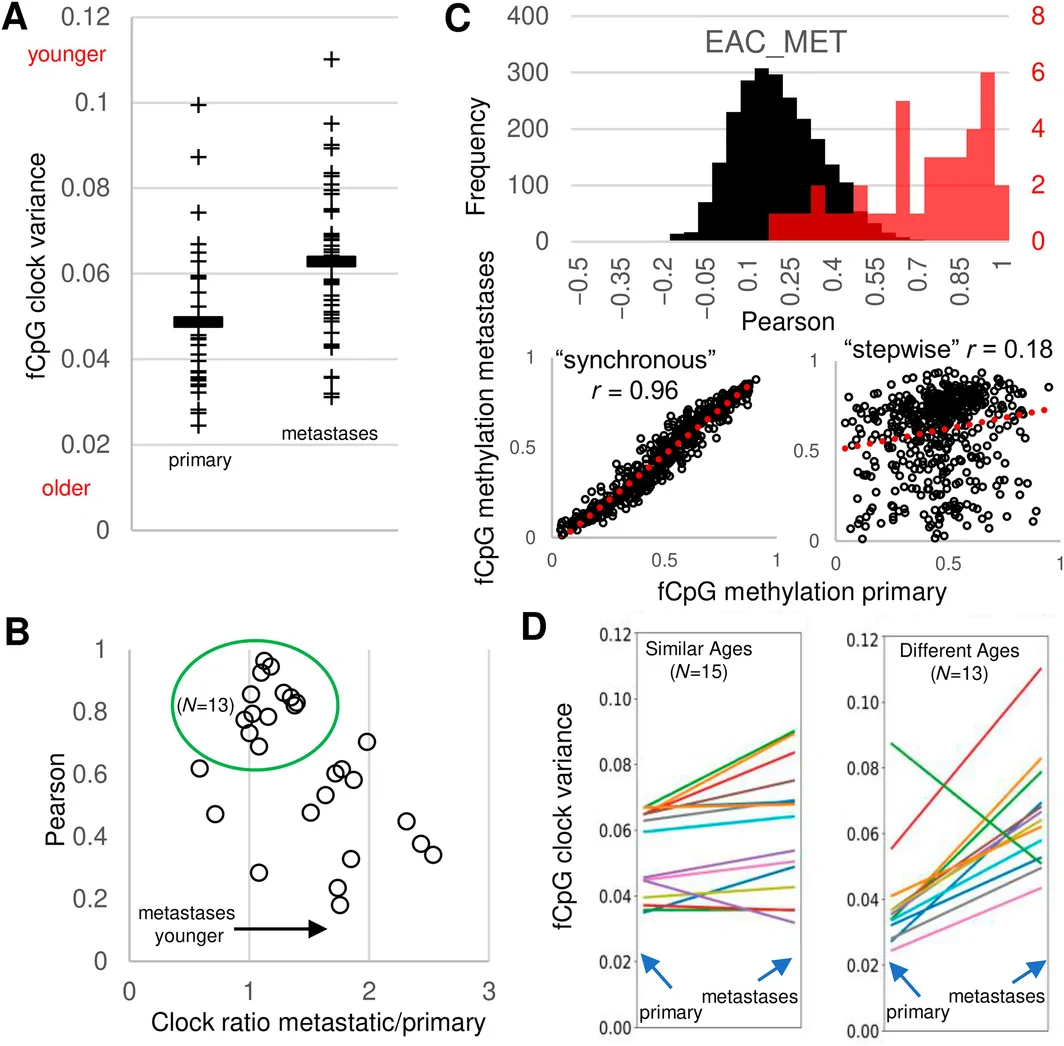
Synchronous and stepwise endometrial adenocarcinoma (EAC) metastases (GSE67116 [11]). (A) Fluctuating CpG (fCpG) variances (mitotic ages) differ between primary and metastatic EACs. Metastatic EAC samples were on average younger than primary cancers (*p =* 0.002). fCpG histograms of each sample are provided in supplementary material, Figure S5. (B) Progression appeared synchronous for 13 of 28 primary and metastatic pairs (circled) with similar mitotic ages (variances) and methylation patterns (Pearson correlation). Similar or synchronous pairs were defined as when metastases/primary fCpG variance ratios were near one (0.75–1.26) and Pearson correlation was greater than 0.6. (C) fCpG methylation patterns were poorly correlated between samples from different individuals, consistent with random fCpG states in progenitor cells at the start of growth. In contrast, primary‐metastatic pairs exhibited a wide range of correlations. Higher correlations are more consistent with synchronous metastasis, whereas lower correlations are more consistent with stepwise progression, particularly when divergence occurs later during growth and when metastases arise from a small number of cells. (D) Mitotic ages (fCpG variances) were similar for 15 of 28 pairs and metastases were much younger than their primary tumors for 12 pairs. There was one example in which the metastasis appeared older than its primary tumor.

The EAC fCpG clock was next applied to paired primary and metastatic samples (GSE67116 [11]). Patients with metastatic EAC have much poorer outcomes, and the timing of metastasis is uncertain. simulations (Figure 3B) indicate that the fCpG clock can infer when metastasis occurs. On average, metastatic tumors were younger than their primary tumors (Figure 5A). Like the adjacent hyperplasia and EAC samples, about half of the metastases (13 of 28) appeared to be synchronous because they had similar mitotic ages and correlated fCpG patterns (Figure 5B). Other metastases appeared to arise stepwise, either because their fCpG methylation was less correlated with their primary tumor (Figure 5C), or because their mitotic ages were much younger than their primary tumors (Figure 5D). One metastatic sample was inconsistent with stepwise progression because the metastasis appeared older than its primary tumor (Figure 5D). Thus, metastases may occur both early or late during EAC progression, consistent with the heterogeneous metastatic phylogenies inferred from sequencing studies [14].

### Immunoediting in older TCGA EACs

**Figure 6:**
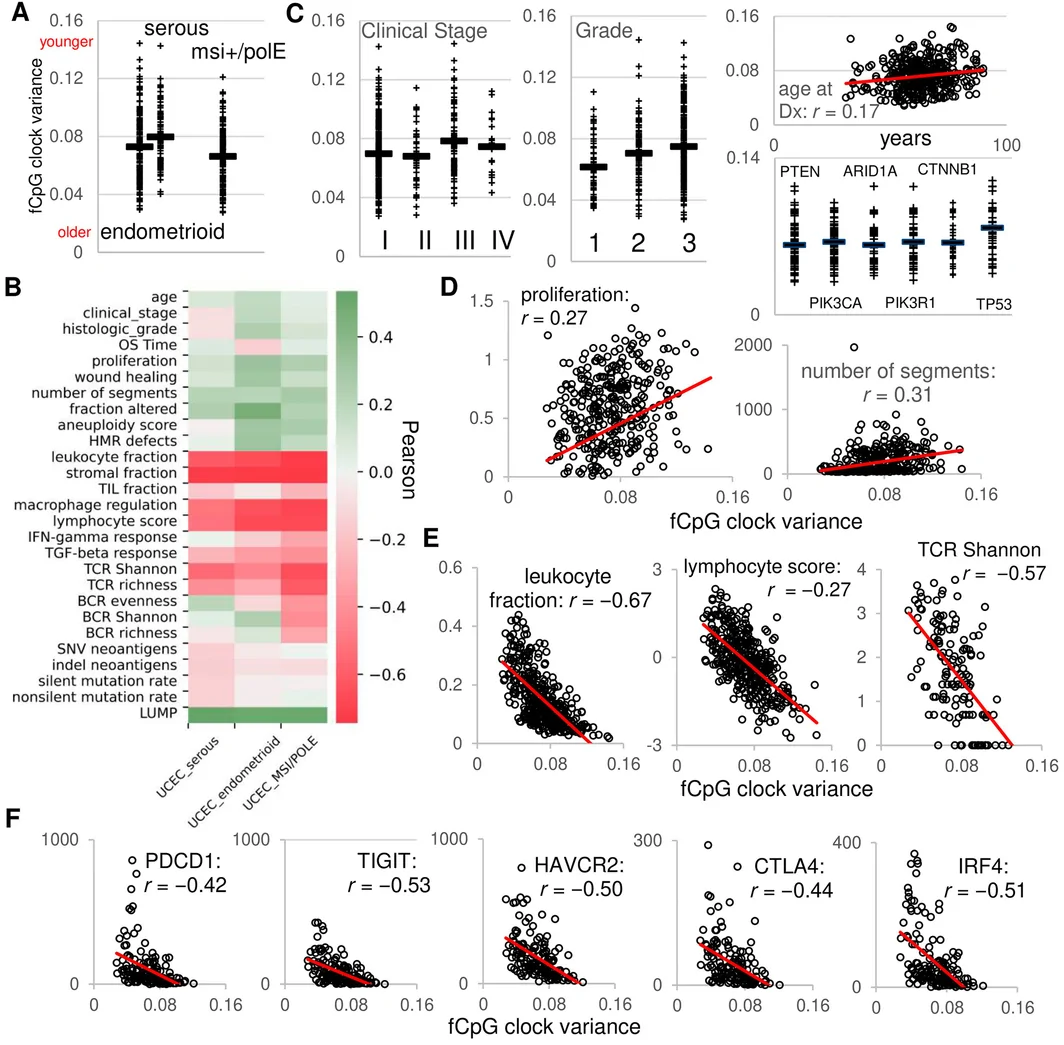
The Cancer Genome Atlas endometrial adenocarcinoma (TCGA EAC) covariate analysis. (A) Fluctuating CpG (fCpG) clock variances vary between TCGA EACs, with serous EACs on average younger than endometrioid (*p* = 0.014) or microsatellite instability‐positive (MSI)+/polE subtypes (*p* < 10^−5^). MSI+/PolE subtypes were significantly older than endometrioid subtypes (*p =* 0.0065). (B) Heatmap of correlations between fCpG clock variances and multiple biological annotations from Thorsson *et al* [12] reveals differences between young and older EACs. Younger EACs had more proliferation and aneuploidy, whereas older EACs showed more evidence of immune surveillance. (C) EAC (all subtypes) mitotic ages did not correlate strongly with tumor grade or stage, age at diagnosis, or with the presence of common somatic mutations including *TP53*. (D) Younger EACs had more proliferation and greater aneuploidy scores (all subtypes, *p* < 10^−5^). The *y*‐axis scales are specific to the outcomes generated in ref. 12. (E) Older EACs had more leukocyte and lymphocyte infiltration scores and greater TCR diversity (all subtypes, *p* < 10^−5^). The *y*‐axis scales are specific to the outcomes generated in Thorsson *et al* [12]. (F) Older EACs showed higher expression of immune checkpoint genes associated with T‐cell exhaustion (MSI+/POLE subtype, *p* < 10^−5^). The *y*‐axis scales are specific to the outcomes generated in Thorsson *et al* [12]. For endometrial and serous subtypes, Pearson was −0.22 and −0.25 for PDCD1, −0.39 and −0.37 for TIGIT, −0.37 and −0.32 for HAVCR2, −0.22 and −0.36 for CTLA4, −0.21 and −0.37 for IRF4.

The fCpG clock was next applied to TCGA EACs, subdivided into mutation‐rich microsatellite instability‐positive (MSI+)/POLE, and serous and endometrial subtypes. Inferred mitotic ages were different between tumors (Figure 6A) and serous EACs were on average younger, consistent with their more aggressive biology. By TCGA subtyping, copy number high (serous‐like) EACs were significantly younger than copy number low (no specific molecular profile, NSMP), MSI+, or POLE EACs (supplementary material, Figure S1).

TCGA cancers have been uniformly annotated with standard algorithms that stratify biological differences based on multiple measurements like RNA‐seq [12]. This large dataset was correlated with EAC mitotic ages to determine why cancer ages may differ (Figure 6B). EAC mitotic ages did not segregate well by common clinical metrics because there were minimal differences with respect to survival, age at diagnosis, stage, grade, or mutation profiles (Figure 6B,C).

Functional differences were present between young and older EACs. Young EACs had greater proliferation, wound healing, and aneuploidy scores (Figure 6B,D). Older EACs had greater evidence of immune surveillance with more immune and stromal cell infiltration, higher macrophage regulation, higher lymphocyte scores, and greater IFN‐γ and TGF‐β responses (Figure 6B,E). Although older EACs did not consistently have greater inferred neoantigen burdens (which may reflect the poor performance of neoantigen prediction algorithms [15, 16]), older EACs had greater TCR Shannon diversity and richness (Figure 6B,E), which may indicate chronic immune stimulation [17, 18].

T‐cell antitumor immunoediting is often directed at clonal somatic mutations [19], and therefore immune‐mediated cell losses could occur throughout growth (Figure 1A). If cell losses during growth are due to immunoediting, a prediction is that the TMEs of older cancers should also be ‘older’ because chronic immune stimulation typically leads to T‐cell exhaustion [20, 21, 22, 23]. Consistent with T‐cell exhaustion [24, 25, 26], immune checkpoint gene expression was higher in older EACs, particularly the MSI+/POLE subtype (Figure 6F), albeit immune cell exhaustion is better measured with single cell data. Older EACs may require more divisions because of immunoediting and may grow to detectable sizes only after chronic immune stimulation leads to T‐cell exhaustion.

### Leukocyte contamination in older cancers

A potential limitation of this analysis is that the more unimodal and more diverse fCpG methylation of older cancers could, in principle, reflect reduced tumor cell purity rather than increased mitotic ages as older cancers tend to have lower tumor cell purity (LUMP [13]) and higher levels of leukocyte and stromal infiltration (Figure 6B,E). To mitigate this risk, only samples with greater than 60% tumor cell purity were included in the analysis. Also, T‐cell and whole blood methylation at the fCpG sites are skewed towards 0% or 100% methylation (supplementary material, Figure S2). Therefore, their contributions to average methylation would tend to decrease rather than increase inferred mitotic ages. Nonetheless, average methylation reflects contributions from all sampled cells, and as such, mitotic ages are inherently less certain for samples with lower tumor cell purity.

## Discussion

The number of divisions needed for cancers to reach detectable size likely varies, and understanding why some tumors require many more divisions may reveal mechanisms that naturally restrain cancer growth. Mitotic age reflects net growth (cell divisions minus cell loss), rather than the time since tumor initiation. Cancers can differ in their mitotic ages only if tumor cell death occurs during growth. Cell death is common in tumors [27], and may be modulated by the TME, with increased loss from insufficient sustainment or from immunoediting [1].

Here we show that EAC mitotic ages vary widely, with striking differences in TME features between younger and older tumors. Growth restraint likely results from multiple mechanisms that may be imposed during different phases of tumorigenesis. At the time of clinical detection when tumors are large, tumor growth is generally slow (often described as Gompertzian [28]). Growth limitations at this stage likely reflect inadequate TME sustainment, and such late size constraints are therefore expected to operate similarly across cancers of all mitotic ages.

In contrast, our data suggest that immune predation much earlier in tumorigenesis, when tumors are small, is a key factor distinguishing younger and older EACs. Early immune‐mediated cell loss increases cell turnover, requiring additional divisions for tumors to eventually reach detectability, thereby inflating mitotic age without producing larger tumors. Thus, while both younger and older cancers are likely constrained by TME sustainment limitations late in growth, older cancers appear to have experienced additional growth restraints early in tumorigenesis due to immunoediting.

Several observations validate the fCpG clock. First, single‐cell cloning studies experimentally demonstrate that new expansions initiated from single cells show the expected ‘W‐shaped’, high‐variance fCpG distributions. Next, adjacent hyperplasia and EAC samples had similar fCpG‐derived mitotic ages, consistent with orthogonal sequencing data that inferred synchronous progression. Likewise, mitotic ages of primary and metastatic EACs aligned with either synchronous or stepwise spread, with only one case in which the metastasis appeared older than its primary tumor. fCpGs also function as lineage barcodes: synchronous growth is reflected by both similar mitotic ages and high correlations of fCpG methylation patterns across regions. Together, these observations provide internal and external validation that the fCpG clock can robustly infer relative mitotic ages.

Correlations with TCGA clinical, sequencing, methylation, and RNA‐seq metadata indicate that immune surveillance restrains EAC growth. Younger cancers require fewer divisions to become detectable, consistent with relatively unimpeded growth and limited immune cell infiltration. By contrast, older cancers have more immune cell infiltration, with evidence of chronic immunoediting manifested by greater TCR Shannon diversity and richness and elevated markers of T‐cell exhaustion. Cancer mitotic ages may therefore reflect a balance between cell divisions and immune‐mediated losses, and older cancers may become detectable only after chronic immune stimulation culminates in T‐cell exhaustion. Potentially, the immune system may recognize and outlast some nascent tumors, successfully eliminating them, consistent with the long‐held idea of Burnet and Thomas [29] that many human tumors are naturally eradicated by immune surveillance. Greater immune cell infiltration was also observed in mitotically older breast cancers [30], supporting a broader role for immunoediting in limiting net growth across tumor types.

Weaknesses of this study stem from the inherent inferential nature of molecular clocks. Experimental clock calibration is difficult because there are no collections of human cancers with well‐documented mitotic ages and age estimates depend on many untested assumptions. We present no direct evidence that EAC mitotic ages vary or on validation of the inferred relative age rankings. We cannot convert mitotic age to calendar ages and fCpG clocks appear to be cell type specific and cannot be easily translated between cancer types. We do not understand the mechanism underlying oscillating or balanced fCpG methylation and demethylation. These weaknesses limit observations to correlations rather than direct evidence.

Despite these limitations, we have tested our clock using datasets with orthogonal ancestral or biological information that render certain age estimates implausible, providing indirect validation. Its precision is uncertain and may only be able to reliably distinguish between very young and very old tumors. Similar tissue‐specific clocks have been used to analyze normal tissues [6], breast cancers [30], and leukemias [6, 31]. The fCpG clock measures average sample methylation, and methylation contributions from non‐tumor cells could confound age estimates, although infiltrating blood or T cells, commonly present in older cancers, would tend to reduce rather than increase inferred ages. Cancer ages and ancestries can be inferred by many methods [2, 3, 4, 32, 33, 34, 35] and continued efforts to reconstruct human tumor histories by multiple complementary approaches should help identify additional mechanisms that naturally limit tumor growth. Importantly, cancer age is not simply another parameter but a history that can explain and organize diverse biological measurements.

## Author contributions statement

KK provided the initial direction and contributed to data interpretation. DS performed the analysis and wrote the manuscript.

## Supporting information


**Figure S1.** Endometrial adenocarcinoma (EAC) variances by The Cancer Genome Atlas (TCGA) subtypes
**Figure S2**. Bimodal fluctuating CpG (fCpG) methylation of T cells and normal blood
**Figure S3**. Fluctuating CpG (fCpG) histograms of fibroblasts (GSE112877)
**Figure S4**. Fluctuating CpG (fCpG) histograms of synchronous hyperplasia–endometrial adenocarcinoma (EAC) pairs (GSE136791)
**Figure S5**. Fluctuating CpG (fCpG) histograms of primary‐metastases pairs (GSE67116)

**File S1.** Lists of fluctuating CpG (fCpG) clock sites and cancers used in this study

## Data Availability

The data used in this study are available from public databases (Gene Expression Omnibus (GEO https://www.ncbi.nlm.nih.gov/geo/): GSE112877, GSE136791, GSE67116, GSE35069, GSE40279) and TCGA (https://portal.gdc.cancer.gov/). Other data that support the findings of this study are available from the corresponding author upon reasonable request.
