# Supplementary material for: Differences between mitotically old and young endometrial tumors
Source: J Pathol. 2026 Jul 8;270(2):163–73. doi: 10.1002/path.70090 (PMC13576881; doi:10.1002/path.70090)
Supplement: Supplementary file 1 [file PATH-270-163-s001.docx]

**Differences between mitotically old and young endometrial tumors**

K Kim and D Shibata *J Pathol* <https://doi.org/10.1002/path.70090>

**Supplementary Figures S1–S5**

**Supplementary File S1 is provided as a separate Excel file**

**Figure S1. Endometrial adenocarcinoma (EAC) variances by The Cancer Genome Atlas (TCGA) subtypes.** By TCGA subclassification, copy number high (serous-like) EACs were significantly younger (*p* < 10^−6^) than copy number low (no specific molecular profile, NSMP) EACs. Ages of microsatellite instability-positive (MSI+), POLE, and copy number low EACs were similar (*p* > 0.05).

**Figure S2. Bimodal fluctuating CpG (fCpG) methylation of T cells and normal blood.** Older cancers, with more unimodal fCpG methylation around 50%, tend to have more stromal contamination, which may reflect biology (immune surveillance), or potential confounding from stromal cell methylation at the clock fCpG sites. Reference datasets for T cells (GSE35069, average of 6 samples) and whole blood ([GSE40279](https://www.ncbi.nlm.nih.gov/geo/query/acc.cgi?acc=GSE40279), average of 53 samples) indicate their fCpG methylation is bimodal. Thus, contributions of leukocytes to average ‘tumor’ fCpG methylation would tend to increase variance, making samples with lower tumor cell purity appear younger rather than older.

**Figure S3. Fluctuating CpG (fCpG) histograms of fibroblasts (GSE112877).** Each histogram shows the frequency distribution of methylation at the 2,000 fCpG sites for each fibroblast sample. Each histogram has its GSM identification number and then either ‘single’ if the sample is a single cell subclone or ‘bulk’ if the sample is a polyclonal culture. The final annotations are the variances of each distribution, where greater variances indicate relatively younger expansions. Data are from <https://www.ncbi.nlm.nih.gov/geo/query/acc.cgi?acc=GSE112877>.

**Figure S4. Fluctuating CpG (fCpG) histograms of synchronous hyperplasia–endometrial adenocarcinoma (EAC) (GSE136791).** Each histogram shows the frequency distribution of methylation at the 507 fCpG sites for each sample. Each histogram has its identification number from reference 10, the sample phenotype (‘Hyperplasia’ or ‘Carcinoma’), and then the variance of the distribution. Greater variances indicate relatively younger expansions. Data are from <https://www.ncbi.nlm.nih.gov/geo/query/acc.cgi?acc=GSE136791>

**Figure S5. Fluctuating CpG (fCpG) histograms of primary-metastases pairs (GSE67116).** Each histogram shows the frequency distribution of methylation at the 507 fCpG sites for each sample. Each histogram has a patient identification number from reference 11, the sample phenotype (‘Primary’ or ‘Metastasis’), and then the variance of the distribution. Greater variances indicate relatively younger expansions. Data are from <https://www.ncbi.nlm.nih.gov/geo/query/acc.cgi?acc=GSE67116>.
